# Fracture Liaison Service and Its Role in Secondary Fracture Prevention in Malaysia: A Scoping Review

**DOI:** 10.5704/MOJ.2307.001

**Published:** 2023-07

**Authors:** WX Lim, HM Khor, JK Lee, T Ong

**Affiliations:** 1 Department of Medicine, Universiti Malaya, Kuala Lumpur, Malaysia; 2 Department of Orthopaedics, Beacon Hospital, Kuala Lumpur, Malaysia

**Keywords:** fragility fractures, osteoporosis, hospital, fracture liaison service

## Abstract

**Introduction:**

Fragility fractures, which occur after a low-trauma injury, increases with advancing age. Such fracture doubles the life-time risk of sustaining another fracture. This risk is highest in the immediate 18 months after the index fracture. However, most patients do not receive the appropriate risk assessment and intervention to reduce this risk. A coordinated model of care termed Fracture Liaison Service (FLS) has been reported to address this treatment gap.

**Materials and methods:**

This scoping review aims to explore the potential role and delivery of FLS services in Malaysia. Scientific and non-scientific sources relevant to FLS were identified from electronic bibliographic databases, specialist journals and relevant websites. Findings were categorised into themes and presented narratively.

**Results:**

FLS services remain concentrated in the Klang Valley. Even within FLS services, many do not have extensive coverage to risk assess all fracture patients. These services are multidisciplinary in nature where there are links between different departments, such as orthopaedics, osteoporosis expertise, bone densitometry, rehabilitation, falls services and primary care. FLS was able to increase the number of people undergoing fracture risk assessment and treatment. The importance of FLS was highlighted by local experts and stakeholders. Its implementation and delivery are supported by a number of national guidelines.

**Conclusion:**

FLS is central to our national efforts to reduce the impending fragility fracture crisis in the coming years. Continued effort is needed to increase coverage within FLS services and across the country. Training, awareness of the problem, research, and policy change will support this endeavour.

## Introduction

Fragility fractures are fractures that occur after a low-trauma injury, defined as impact from a fall from standing height or less^1^. The incidence of fragility fractures increases with advancing age^2,3^. It is estimated that the global burden of such fractures will continue to increase, very much driven by an ageing population and an increase in the number of people living with osteoporosis^2,4^.

Fragility fractures, such as those affecting the spine, hip, pelvis, wrist and shoulder, are associated with significant disability, dependency and morbidity^3^. Hip fractures, considered the worst fragility fracture, has a one-year mortality as high as 25% and among those that survive up to 60% require assistance with daily living^5^. It also leads to an increase in care cost^6^. It is estimated that under 6000 hip fractures occur annually in Malaysia^7^. The Asian Federation of Osteoporosis Societies (AFOS) predicted that over the next two decades, there will be up to 21,000 hip fractures annually^7^. This represents the highest rate of increase in the number of hip fractures compared to other member states in the AFOS, due to Malaysia’s rapidly ageing society^7^.

Sustaining a fragility fracture doubles the life-time risk of another fracture^8^. However, this risk is not constant over time as it can be as high as five-times in the immediate 18 months after the initial fracture^9^. Most fragility fractures occur after a fall and it indicate underlying osteoporosis^2,3^. Hence, with appropriate assessments and subsequent treatment for a person’s falls risk and bone health, that future fracture risk can be reduced. However, these assessments are not routinely performed after a fracture exposing those that have sustained a fragility fracture to developing another one^5,10^.

To address this secondary fracture prevention gap, a coordinator-based model of care termed Fracture Liaison Service (FLS) has been recognised to be the solution to this^11^. It operates by proactively and systematically identifying adults with a fragility fracture for secondary fracture prevention. Patients are assessed for their falls, osteoporosis and fracture risk. Appropriate treatment is recommended, initiated and followed-up. FLS services have doubled the number of bone mineral density (BMD) assessments, osteoporosis treatment initiated and treatment adherence^12^. This has translated into a 30% reduction in the risk of a subsequent fracture^12^. Therefore, international societies and clinical practice guidelines now strongly recommend FLS as part of any post-fracture care^11,13^.

A secondary fracture prevention gap exists in Malaysia^14-17^. Many do not receive the required assessments and fracture risk management. What remains uncertain is if FLS could be the solution to this local problem, and if so, what should be its implementation and delivery strategy. To address this, a scoping review was performed to examine the range of evidence informing FLS delivery in Malaysia.

## Materials and Methods

This review was exploratory in nature of the potential role and delivery of FLS services in Malaysia. It was envisaged that a significant proportion of the literature surrounding FLS locally would come from various publication sources. This work would also be used as a basis for future FLS development work specific to the Malaysian healthcare context. Hence, a scoping review based on the framework methodology by the Joanna Briggs Institute (JBI) was selected as the choice of evidence synthesis to inform this review^18^.

Scientific and non-scientific sources relevant to FLS were identified. Electronic bibliographic databases identified were PubMed, Google Scholar and the Malaysian Medical Repository. The search was performed from inception till 15.12.2022 using the terms and its relevant synonyms for “fracture prevention”, “fracture liaison service” and “Malaysia”. References from any identified studies would also be screened for further eligible studies. Other sources which formed this study’s search strategy included the Malaysian Orthopaedic Journal’s site which archives non-indexed literature, abstracts from the World Congress on Osteoporosis, Osteoarthritis and Musculoskeletal Diseases (WCO-IOF-ESCEO) published in Osteoporosis International, Google search engine up till the first 10 pages, and related websites (International Osteoporosis Foundation, Fragility Fracture Network, Fragility Fracture Network Malaysia, Malaysian Osteoporosis Society, Malaysian Society of Geriatric Medicine, Malaysian Orthopaedic Association, Malaysian Endocrine and Metabolic Society and Malaysian Society of Rheumatology). Any study regardless of design and type of article that described FLS or a co-ordinated secondary fracture prevention programme in Malaysia was included.

Data from the publication identified will be extracted and categorised into themes that covered the scope and coverage of FLS in Malaysia, its organisation and service delivery, outcomes, and related policy documents. Data analysis and reporting of findings were narrative based on the set themes. Gaps in the literature that may require further research were also highlighted.

## Results

### Coverage of Fracture Liaison Service (FLS)

Coverage of FLS services have only been reported by the International Osteoporosis Foundation Capture the Fracture (IOF CtF) on its website^19^. There are currently 12 FLS services in Malaysia mapped to it. Ten of these services are based at public hospitals and two in the private sector. Five of them are based in the Klang Valley (i.e. around the capitol of Malaysia). The first FLS was established in 201720. The IOF CtF also rated individual FLS services to either Gold, Silver, Bronze or Blue based on the extend, coverage and proportion of patients with fragility fractures captured for further fracture risk assessment. There is only one Gold-star rated FLS in Malaysia at this moment in time. Although other FLS sites likely exist outside the IOF CtF map, this study was unable to list them.

### Organisation and delivery of the FLS service

Only one FLS in Malaysia has reported on the composition of its FLS team^20^. In this private hospital based FLS, the team consisted of a FLS clinical lead (an orthopaedic surgeon), a FLS coordinator, rehabilitation services, dietician, and the wider medical team from orthopaedic surgery and internal medicine. Family doctors were recruited as part of the patient care services.

Two sites reported on its FLS patient eligibility. Individuals aged 50 years and above who experienced a fragility fracture (defined as fractures following a fall from standing height or less) involving the hip, wrist, upper arm, pelvis or spine are included in the program^20,21^. These patients would be from both the inpatient and outpatient setting.

The FLS service would provide a multicomponent intervention. Individuals would have imaging to diagnose osteoporosis and its severity, offered medication (oral or parenteral) to prevent further fractures and improve bone density, and be educated on adopting healthy lifestyle choices for their bone health. After discharge, these patients will be followed-up for up to one year after the fracture^20,21^. The care will be coordinated by the FLS coordinator. The FLS Coordinator will contact the patient / family members to inquire about the patient's compliance with anti-osteoporosis medications, functional status, and any subsequent falls or challenges the patient encounters at home^20^. The FLS service has now been incorporated into standard care for all patients admitted with a fragility fracture. An important part of this hospital’s FLS is creating awareness of the importance of a FLS service. Public awareness was also listed as an aim of the service.

### Impact of FLS

Only one public hospital FLS reported on the impact of their FLS service. Since the FLS service was implemented, 3-4 times more patients have had their bone mineral density (BMD) assessed and osteoporosis medication prescribed^21-23^. At six months and one year, 83% and 82.3% were still persisting with their osteoporosis medication^23^. The common reason reported for patients not persisting included patients’ decision on the matter (35.7%), followed by financial issues (29%)^23^. Other contributing factors were loss to follow-up, side-effect of medication and transportation to collect the medication.

### National consensus view on FLS

There have been consensus papers and local clinical guidelines that have been published supporting FLS services and its expansion. Local experts have reported that FLS in Malaysia has shown a lot of promise for improving bone health^24,25^. Its expansion will be supported by the push by specialist societies and national clinical practice guidelines advocating for it^25^. Additionally, several national guidance has recommended FLS services to improve the detection and treatment of individuals at risk of another fragility fracture. These guidelines include the Malaysian Osteoporosis Society management of osteoporosis clinical practice guideline^26^, the Malaysian Society of Geriatric Medicine (MSGM) position statement on falls and fragility fractures^27^, and the Fragility Fracture Network Malaysia (FFNM) FLS framework^28^.

These guidelines highlighted the role of the FLS service and underpinning the success of this service delivery is a multidisciplinary approach. Coordination with other healthcare professionals and services including emergency departments, orthopaedic units, rehabilitation providers, bone densitometry services, falls clinics, primary care, and clinicians with osteoporosis expertise is key^26-28^. Central to it, is the engagement with the person with the fracture and their caregiver^27^. Initiating and sustaining a FLS requires management leadership, organisation support, stakeholder engagement and an adequate investment of resources (infrastructure, staff and support systems). The guidelines advise upscaling services from focusing on hip fracture, to inpatient non-hip fractures, outpatient fractures and finally incidental vertebral fractures^26^.

The Fragility Fracture Network Malaysia (FFNM) was established to improve the care for people with fragility fractures^29^. A key objective of this organisation is to prioritise secondary fracture prevention and promote the role of FLS. To achieve this, FFNM has further collaborated with the Malaysian Orthopaedic Association, Malaysian Osteoporosis Society and the Osteoporosis Awareness Society of Kuala Lumpur and Selangor^29^. Additionally, FFNM has taken this a step further with the publication of a FLS framework which covers the case for a FLS service in individual hospitals, how to set up a service, a minimum common dataset to audit service delivery and monitoring^28^.

## Discussion

This scoping review has explored the current state with FLS services in Malaysia. There are 12 FLS services mapped to IOF CtF, and many are based in urban Klang Valley^19^. Such a narrow coverage means that the vast majority of Malaysians do not have access to a FLS service. Most of these existing FLS still have room to increase the patient identification and coverage of their service. There may be other FLS services not mapped and the number of actual FLS could be higher. Regardless of services being mapped or not, recognising individual FLS sites help stakeholders identify where the gaps are in service provision, a means of networking and peer-support, and eventually the possibility of national audit which has been shown to drive the quality of FLS services^30^.

The majority of people after a fracture do not have the required assessment to prevent another fracture. A university hospital reported that only one-third of patients after a fracture had a plan for BMD assessment, but only 64% attended. A quarter of these patients were initiated on osteoporosis treatment and half persisted with it after 12 months^16^. This gap will widen as the number of people with fractures will increase due to the change in ageing demographics that is being seen in Malaysia^7^. FLS service has been shown to be an effective intervention in other countries^12^ and in one local FLS service there have reported a 2 to 3-fold increase in BMD testing and treatment initiation^21,22^.

Osteoporosis affects 1-in-6 women and 1-in-9 men^31^. Half of adults have osteopenia, a pre-osteoporosis stage^31^. The need to expand FLS coverage nationally becomes even more pressing when reports such as the one by AFOS highlighted that Malaysia will have the highest rate of hip fracture increase among all its member states. Half of people with hip fractures have sustained a previous fracture^32^. A fracture itself also doubles the life-time risk of another fracture^8^. Fractures themselves are not benign conditions. They are associated with dependency, disability and mortality^33^. Local experts and specialist national societies have been consistent with their recommendation that FLS is the solution to effective fracture secondary prevention^24-28^. Although the impact of FLS service in Malaysia has not been extensively reported, there is no reason why clinical benefits seen elsewhere would not translate into Malaysia’s population. The impact of a FLS program across Singapore’s public hospitals has seen improvement in secondary fracture prevention care^34^. Health economic analysis conducted in countries such as Australia, Canada, United Kingdom, United States and Japan have demonstrated the FLS cost-effectiveness^35^.

This review has highlighted the gap in Malaysia’s FLS provision and paucity of locally published literature. This is on the background of an ageing population and a predicted rise in the number of fragility fractures. Maintaining status quo is not an option, especially when there is an evidence-based clinical and cost-effective intervention to address the problem. FLS ensures that all patients are adequately risk-assessed for their risk of further fractures, initiated on appropriate management and persist with their treatment. To push this agenda, the public and healthcare practitioners need to be more informed about the impact of osteoporosis and fragility fractures. FLS champions the will to engage with local hospitals to establish and support the implementation of FLS services. Reporting of such services and its impact will help to build the case for those more reliant on local examples to drive the change. Research in this area needs to be encouraged to support its implementation and adoption. Specialist societies need to continue to advocate for this to be part of national policy, utilising recommendations made by national guidelines.

## Conclusion

Fragility fractures have significant health and care implications. FLS services will be the key to address the secondary fracture prevention gap in Malaysia. Existing services need to be consolidated and new FLS developed to broaden its catchment area across Malaysia. A multipronged approach is needed to deliver this.
